# Penetrating Injury to the Upper Extremity

**Published:** 2013-01-21

**Authors:** Ruth N. Celestin, Ramazi Datiashvili

**Affiliations:** New Jersey Medical School, Division of Plastic Surgery, University of Medicine and Dentistry, NJ

**Figure:**
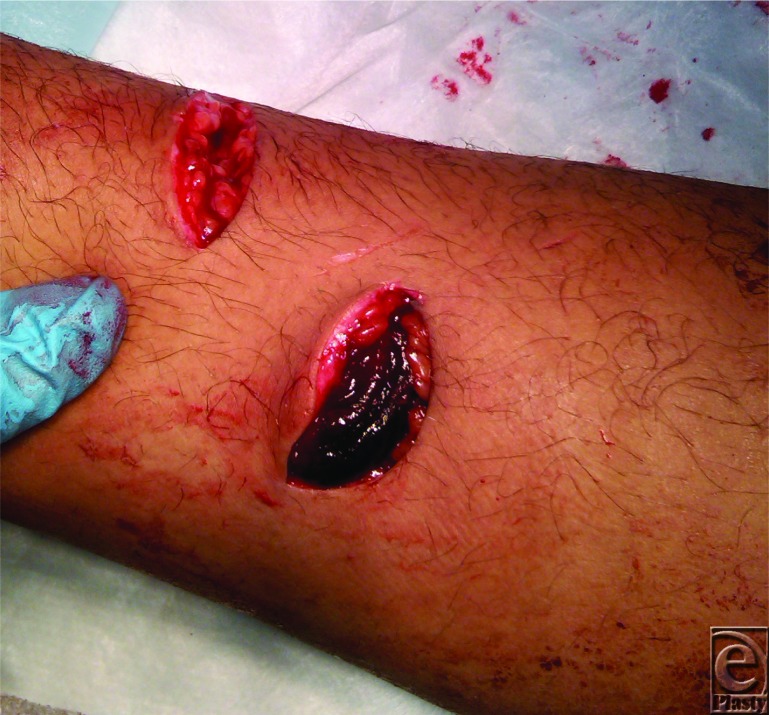


## DESCRIPTION

A 16-year-old right-hand-dominant adolescent boy presents to the emergency department with multiple lacerations to the right forearm after falling through a glass table with an outstretched hand. He has pulsatile bleeding from the proximal wound and normal sensorimotor examination of the right hand.

## QUESTIONS

1. **What structures are most likely injured in this patient?**
2. **List 4 signs of arterial injury in an upper extremity.**
3. **What type of surgical treatment is indicated?**

## DISCUSSION

The presence of brisk bleeding from the upper extremity laceration in this patient coupled with a normal sensorimotor examination of the right hand indicates a high likelihood of isolated arterial injury. Physical examination should include palpation for pulses; however the presence of a radial pulse in the upper extremity does not rule out proximal injury to the vessel. This is because of the rich collateralization of blood flow around the shoulder and elbow. Conventional angiography may be of use in evaluating patients where the level of injury is unclear, but it is rarely needed.

Reliable indicators of extremity arterial injury include active or pulsatile hemorrhage, pulsatile or expanding hematoma, diminished or absent pulses, and evidence of ischemia on clinical examination.

Conservative nonsurgical treatment of these injuries remains controversial. In surgical treatment, incisions in the extremities are designed longitudinally over the injured vessel and are extended proximally and distally as necessary.

While some studies demonstrate the development of claudication, extremity weakness, and cold intolerance, overall review of the literature would suggest that ligation of an isolated radial or ulnar artery is appropriate and well tolerated. When both the arteries are injured, the repair of ulnar artery takes precedence because of its role as the dominant arterial supply to the hand.

The type of vascular repair depends on the nature of the arterial damage. Sharp injury often lends itself to primary repair. Primary repair with an end-to-end anastomosis is performed with a running or interrupted nonabsorbable monofilament suture. If a large gap prevents tension-free repair, reversed saphenous or cephalic-vein autogenous interposition grafts must be used for reconstruction.

All repairs must be covered with viable soft tissue. Splinting of the extremity is useful for preventing disruption of the repair with patient movement. It is also important to avoid compression of the vascular repair. Intraoperative completion arteriography is recommended, and palpable distal pulses should be documented after repair. Venous injury to the upper extremity rarely requires repair because the collateral network is extensive. Ligation of the venous injury is typically well tolerated.
